# Generation of *SNCA* Cell Models Using Zinc Finger Nuclease (ZFN) Technology for Efficient High-Throughput Drug Screening

**DOI:** 10.1371/journal.pone.0136930

**Published:** 2015-08-28

**Authors:** Warunee Dansithong, Sharan Paul, Daniel R. Scoles, Stefan M. Pulst, Duong P. Huynh

**Affiliations:** Department of Neurology, University of Utah, 175 North Medical Center Drive East, 5th Floor, Salt Lake City, Utah, 84132, United States of America; UCL Institute of Neurology, UNITED KINGDOM

## Abstract

Parkinson’s disease (PD) is a progressive neurodegenerative disorder caused by loss of dopaminergic neurons of the substantia nigra. The hallmark of PD is the appearance of neuronal protein aggregations known as Lewy bodies and Lewy neurites, of which α-synuclein forms a major component. Familial PD is rare and is associated with missense mutations of the *SNCA* gene or increases in gene copy number resulting in *SNCA* overexpression. This suggests that lowering *SNCA* expression could be therapeutic for PD. Supporting this hypothesis, *SNCA* reduction was neuroprotective in cell line and rodent PD models. We developed novel cell lines expressing *SNCA* fused to the reporter genes luciferase (*luc*) or *GFP* with the objective to enable high-throughput compound screening (HTS) for small molecules that can lower *SNCA* expression. Because *SNCA* expression is likely regulated by far-upstream elements (including the NACP-REP1 located at 8852 bp upstream of the transcription site), we employed zinc finger nuclease (ZFN) genome editing to insert reporter genes in-frame downstream of the *SNCA* gene in order to retain native *SNCA* expression control. This ensured full retention of known and unknown up- and downstream genetic elements controlling *SNCA* expression. Treatment of cells with the histone deacetylase inhibitor valproic acid (VPA) resulted in significantly increased *SNCA-luc* and *SNCA*-*GFP* expression supporting the use of our cell lines for identifying small molecules altering complex modes of expression control. Cells expressing *SNCA-luc* treated with a luciferase inhibitor or *SNCA* siRNA resulted in *Z*’-scores ≥ 0.75, suggesting the suitability of these cell lines for use in HTS. This study presents a novel use of genome editing for the creation of cell lines expressing α-synuclein fusion constructs entirely under native expression control. These cell lines are well suited for HTS for compounds that lower *SNCA* expression directly or by acting at long-range sites to the *SNCA* promoter and 5’-UTR.

## Introduction

Parkinson’s disease 1 (PARK1) is an autosomal dominant disorder caused by missense mutations and multiplications of the *SNCA* gene, encoding α-synuclein [1–3]. Although missense mutations are rare events, duplications and triplications of the *SNCA* gene [1–9] are found in both familiar and sporadic PD, and have been linked to more than 30 families with PD and parkinsonism [5]. The common occurrences of *SNCA* genomic multiplications point to the importance of gene dosage and overexpression of wildtype α-synuclein in causing neurodegeneration in α-synucleinopathies [3]. These observations were in line with data showing neuronal toxicity in cell and animal models of α-synuclein overexpression [10–17]. Elevated levels of wild type α-synuclein in patient brains or patient-derived cell lines were also observed in sporadic PD [18–20] and in familial PD caused by mutations in *PARK2* [14,16,21], *GBA* [10], and *LRRK2* [12]. These observations support the widely held hypothesis that elevated levels of α-synuclein cause death of dopaminergic neurons in PD. Reducing the levels of α-synuclein was neuroprotective in several studies of cellular and animal models of α-synucleinopathies [14,22–24]. Furthermore, *SNCA* knock-out mouse models showed increased dopamine release with paired stimuli or elevated Ca^2+^, but exhibited no PD phenotypes [25,26]. Therefore, reducing the level of α-synuclein likely delays the onset of PD phenotypes with fewer risks to the recipients. However, specific compounds that reduce the expression and levels of endogenous α-synuclein for therapeutic application have not been identified. One impediment to identifying such compounds is the lack of cell line models that express *SNCA* in its proper genomic context.

Expression control is complex for *SNCA*, involving control elements that are located far upstream of the *SNCA* transcriptional start site, a complicated structure of repeats in the promoter controlling *SNCA* transactivation and epigenetic expression control. One prominent feature of the *SNCA* promoter is NACP-REP1, a regulatory element consisting of a complex structure of dinucleotide CT, TA, and CA repeats, flanked by two domains that enhance *SNCA* expression [27,28]. The NACP-REP1 is located at 8852 bp upstream of the *SNCA* transcription start site [29]. Polymorphisms at the NACP-REP1 region regulate *SNCA* expression, and dinucleotide polymorphisms at the NACP-REP1 locus were associated with Parkinson’s and Alzheimer’s Diseases [30,31]. In addition, a decrease in hypermethylation of the *SNCA* promoter CpG island has been observed in sporadic PD [32,33], and promoter CpG hypomethylation was correlated with increased α-synuclein expression in a HEK293 cell model [33]. Expression control by far upstream regions can involve complex chromatin loops and epigenetic modifications [34,35]. We reported the generation of cell lines that preserve the entire promoter regulator networks controlling *SNCA* expression for HTS of small compounds that alter the interactions of the distal elements with the *SNCA* promoter.

Because of the complexity of *SNCA* expression control and great length of the *SNCA* promoter, we employed genome editing to introduce reporter genes downstream of the *SNCA* locus to create cell line models for identifying *SNCA* inhibitory compounds. In the present study, we utilized zinc finger nuclease (ZFN) genome editing [36–38] to knock-in the desired reporter gene sequences into the *SNCA* locus. This approach is advantageous to transfecting expression plasmids containing the targeted cDNA due to high integration efficiency and rare integration at off-target sites [36,38]. Additionally, this approach is more likely to report expression from the gene of interest (*SNCA*) since the entire expression control mechanism is retained. The objective of this study was to generate SH-SY5Y neuroblastoma cell lines edited to express α-synuclein fusions to luciferase or green fluorescent protein (GFP) under the regulation of the complete, native *SNCA* promoter/enhancer system, and to demonstrate the utility of these cell lines for compound screening.

## Materials and Methods

### Ethics Statement

No animal or human participants were used in this research.

### Cell Lines

SH-SY5Y neuroblastoma (ATCC, CRL-2266) and HEK-293 (ATCC, CRL1573) cell lines were purchased from American Type Culture Collection (Manassas, VA).

### *ATXN2* cell lines

The HEK293 cell line expressing *ATXN2-luc* was previously established by our lab [39]. Briefly, HEK293 cells were stably transfected with plasmid pGL2-5A3 to create cell lines H2 and S2, respectively. Plasmid pGL2-5A3 includes -1062 to +660 of the *ATXN2* gene ending on the first CAG of the CAG tract, upstream of the luciferase gene. The luciferase start codon was mutated to CTG to fuse the ataxin-2 fragment with luciferase. The luciferase gene is followed by 1019 bp of *ATXN2* 3’-UTR and downstream sequence (+4098 to +5116). *ATXN2* bp positions are relative to the transcription start site (TSS) as described in Scoles et al. [39]. Selection was accomplished using hygromycin.

### ZFN genome editing

**Fig 1:**
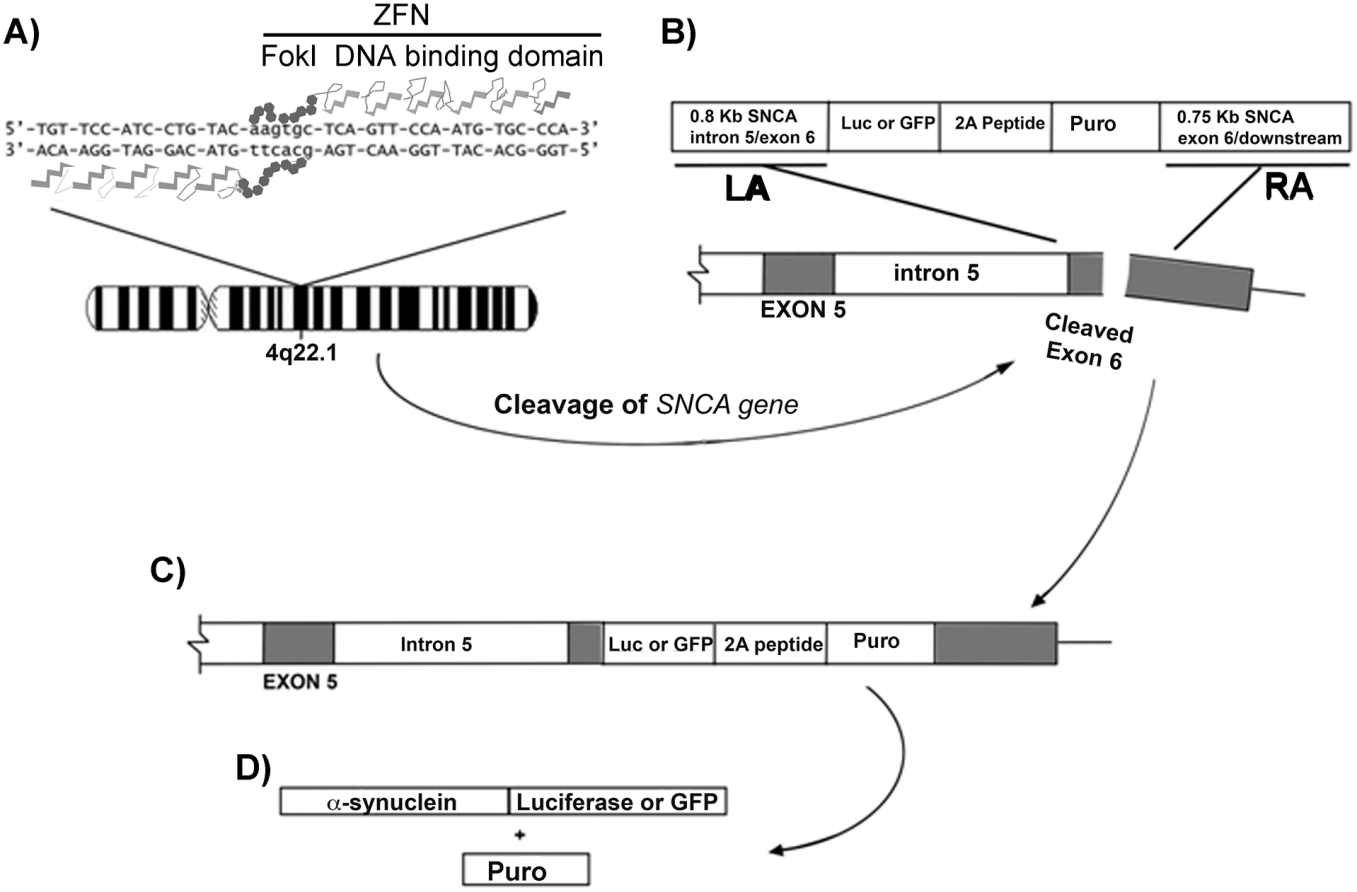
Diagram of Zinc Finger Nuclease (ZFN) gene targeting. SH-SY5Y cells were transfected simultaneously with left and right ZFN plasmids **(A)** and a donor DNA **(B)** plasmid. The ZFN consists of a DNA binding and a FokI nuclease domain. Cleavage of the target DNA by ZFN **(B)** stimulates homologous recombination with the donor DNA. The result is insertion of either the *luc-2A-Puro* or the *GFP-2A-Puro* gene cassette **(C)** in-frame downstream of the *SNCA* gene, leaving the *SNCA* 3’-UTR intact. The donor DNA included a 2A peptide that is cleaved by endogenous peptidase leading to two protein products including the tagged α-synuclein-luciferase (α-syn-luc) or α-synuclein-GFP (α-syn-GFP) and the product of the puromycin (Puro) resistance gene **(D)**.

A pair of *ZFN-FokI* plasmids was custom-made by Sigma Aldrich. We designed and constructed the donor plasmid, which consists of the *GFP-2A-puromycin (GFP-2A-Puro)* resistant or *luciferase-2A-puromycin (luc-2A-Puro)* gene cassette flanked by ~800 bp sequences up- and downstream of the ZFN-FokI cleaved site in the *SNCA* gene (Fig 1 and S1 Fig). The *ZFN-FokI* plasmid and donor plasmid were cotransfected into SH-SY5Y cells, selected by 10 μg/ml puromycin, and confirmed by RT-PCR, qPCR, Western blots, *SNCA* siRNA, and VPA treatments.

### Construction of the right and left ZFN-FokI expression plasmids

To generate cell lines expressing full-length α-syn-luc (α-synuclein-luciferase) and α-syn-GFP (α-synuclein-green fluorescent protein) fusion proteins under the regulation of the native regulatory elements, we used the Zinc Finger Nuclease (ZFN) genome editing method [36]. The ZFN plasmids that we produced create a double strand break (DSB) at the *SNCA* locus that in the presence of a customized donor DNA, directly inserts a reporter gene (*luc* or *GFP*) in frame with the last codon of the *SNCA* gene (Fig 1). We employed a pair of custom-made *ZFN-FokI* plasmids (Sigma Aldrich) that specifically cleaved at a site 59 bp downstream of the targeted locus (S1 Fig). The ZFN plasmids were verified by Cel-1 nuclease assay using PCR primers flanking the predicted breakage point which generated an uncut PCR product of 329 bp (S1 Fig). Cel-1 nuclease cleaved the mismatched short nucleotide sequence formed by random repairing of the breakage point producing 2 bands of 195 and 134 bp (S1 Fig, Sigma Aldrich). Since the DNA binding sequences of the left and right ZFN binding domains were known, the cleavage site (aagtgc) of the ZFN was determined to locate at 59 bp downstream of the TAA stop codon of the *SNCA* gene (S1 Fig).

### Construction of the *SNCA-2A-GFP* donor plasmids

We created donor plasmids containing the *GFP* reporter gene and puromycin resistant gene (*Pac* or “*Puro*”) flanked by the 5’- and 3’- sequence of the *SNCA* gene (Fig 1), located immediately on either side of the target site. The *SNCA-GFP-2A-Puro* donor plasmid consisted of the *GFP* cDNA ligated in-frame with the *SNCA* gene, followed by a short oligonucleotide encoding the 2A peptidase signaling peptide, and *Puro* cDNA. The *GFP-2A-Puro* cassette sequence was derived from the p*OCT4-GFP-2A-Puro* Donor 3 plasmid [36] (Addgene, Plasmid #22211). The insert cassette was flanked by an 800 bp upstream (Fig 1, Left arm, LA) and 750 bp downstream sequence (Fig 1, Right arm, RA) of the target site of the *SNCA* gene. The initiation codon (ATG) of the *GFP* cDNA was replaced with CTG by inserting a NotI restriction enzyme in place of the NcoI site to prevent expression of free GFP.

### Construction of the *SNCA*-*Luc-2A-Puro* donor plasmid

A similar strategy to that used for *SNCA-GFP-2A-Puro* was used to generate the *SNCA-luc-2A-Puro* donor plasmid, with the difference being that the *GFP* cDNA in the *SNCA-GFP-2A-Puro* plasmid was replaced with the *luc* cDNA to create *SNCA-luc-2A-Puro* donor plasmid. Similar to the *SNCA-GFP-2A-Puro* donor plasmid, the ATG on the *luciferase* (*luc*) cDNA was replaced with CTG to prevent the expression of free luciferase.

### Generation of stable SH-SY5Y cell lines expressing α-syn-GFP or α-syn-luc

To generate SH-SY5Y cell lines expressing α-syn-luc or α-syn-GFP, SH-SY5Y cells were transfected with a cocktail of ZFN and donor plasmids using Invitrogen lipofectamine 2000. The transfected cells were selected with 10 μg/ml puromycin (InvivoGen). Puromycin selected colonies from each positive well were plated in new tissue culture dishes for storage and cell line characterization using RT-PCR, qPCR, Western blots, *SNCA* siRNA, and VPA induction. Three cell lines were created and designated GFP12, Luc6B, and Luc6B-5 for their expression of GFP (GFP12) or luciferase (Luc6B), respectively.

### Valproic acid treatment

Valproic acid (VPA) treatments were performed according to Choi et al [40], Equal numbers of Luc6B cells were plated in 384-well tissue culture plates and grown in DMEM/FBS/puromycin containing the indicated concentrations of VPA for the designated times. Luciferase assays were performed according to the protocol provided in the Promega Bright-Glo Luciferase Assay System Kit (Cat # E2620). VPA was purchased from Sigma Aldrich.

For the GFP12 cell line, 5000 cells were plated in 384-well, plate containing the indicated concentrations of VPA in phenol red free DMEM/10% FBS/10 μg/ml puromycin. After 72 hours, GFP fluorescent intensity was measured using a Beckmann DTX880 plate reader, then MTT viability assays (Promega’s CellTiter96Q kit, cat #5421) were performed. For Western blots and qPCR, equal numbers of GFP12 or Luc6B cells were cultured in six-well dishes overnight. The next day, cells were treated with VPA at 0, 5 or 10 mM for 48 hrs. After treatment, cells were harvested and evaluated by qPCR and Western blotting.

### RT-PCR and quantitative RT-PCR

Total RNA was extracted using the RNeasy Mini Kit (Qiagen Inc., USA) according to the manufacturer’s protocol. DNAse I treated RNA samples were used to synthesize cDNAs using the ProtoScript cDNA First Strand cDNA Synthesis Kit (New England Biolabs Inc., USA). Synthesized cDNAs were used for RT-PCR and qPCR analyses. *GAPDH* amplification was conducted in parallel as an internal control for RNA quality and was employed to evaluate the quality of the reverse transcriptase reactions. Quantitative RT-PCR was performed in QuantStudio 12K (Life Technologies, Inc., USA) with the Power SYBR Green PCR Master Mix (Applied Biosystems Inc, USA). PCR reaction mixtures contained SYBR Green PCR Master Mix and 0.5 pmol primers and PCR amplification was carried out for 45 cycles: denaturation at 95°C for 10 sec, annealing at 60°C for 10 sec and extension at 72°C for 40 sec. The threshold cycle for each sample was chosen from the linear range, converted to a starting quantity by interpolation from a standard curve run on the same plate for each set of primers. All gene expression levels were normalized to the *GAPDH* mRNA levels. Primer pairs designed for RT-PCR and qPCR are listed in (S1 Table).

### Preparation of protein lysates and Western blots

Cellular extracts were prepared by the single-step lyses method [41]. The cells were harvested and suspended in SDS-PAGE sample buffer (2x Laemmli Sample Buffer; Bio-Rad; cat# 161–0737) and then boiled for 5 min. Equal amounts of the extracts were subjected to Western blot analysis to identify wild type α-synuclein, α-syn-GFP, α-syn-luciferase, and actin using antibodies listed below. Protein extracts were resolved by SDS-PAGE and transferred to Hybond P membranes (Amersham Bioscience Inc., USA). After blocking with 5% skim milk in 0.1% Tween 20/PBS, the membranes were incubated with primary antibodies in 5% skim milk in 0.1% Tween 20/PBS for two hrs at room temperature or overnight at 4°C. After several washes with 0.1% Tween 20/PBS, the membranes were incubated with the corresponding secondary antibodies conjugated with HRP in 5% skim milk in 0.1% Tween 20/PBS for two hrs at room temperature. Following three additional washes with 0.1% Tween 20/PBS, signals were detected by using the Immobilon Western Chemiluminescent HRP Substrate (Millipore Inc., USA; cat# WBKLSO100) according to the manufacturer’s protocol. The following antibodies were used throughout the study: α-synuclein mAb [(1:1000), Santa Cruz Inc.; cat# sc-12767 (ab 211)], α-synuclein mAb [(1:1000), Santa Cruz Inc.; cat# sc-58480 (LB509)], GFP mAb [(1:3000), Santa Cruz Inc.; cat# sc-9996] and goat anti-Luciferase [(1:3000), Rockland Immunochemicals]. To control for protein quality and loading, the membranes were re-probed with β-Actin mAb conjugated with HRP [(1:10,000), Sigma Inc, cat# A3858]. The secondary antibodies were anti-mouse IgG-HRP [(1:5000), Vector laboratories; cat# PI-2000], anti-goat IgG-HRP [(1:5000), Vector laboratories; cat# PI-9500]. To improve the detection of wild type α-synuclein, the filters were incubated with 0.4% PFA for 30 minutes right after Western blot transfer to cross-link the wild type α-synuclein to the filter prior to incubation of with α-syn antibody [42].

### Control and *SNCA* siRNA transfection

GFP12 and Luc6B cells were cultured and maintained in DMEM media with 10% fetal bovine serum containing 10 μg/ml puromycin. For siRNA-mediated depletion of α-synuclein and α-syn mRNA, cells were cultured in 6 well dishes overnight and siRNAs; Control siRNA (Santa Cruz Inc.; cat# sc-37007) and α-synuclein siRNA (Santa Cruz Inc.; cat# sc-29619) at a concentration of 100 nM were transfected into GFP12 or Luc6B cells using lipofectamine 2000 (Invitrogen Inc.), according to the manufacturer’s protocol. At five days post-transfection, cells were harvested for Western blot analyses.

## Results

The human *SNCA* gene is predicted to generate 11 isoforms resulting in gene products ranging from 67 aa to 140 aa (ENSEMBL.ORG, GENE CODE: ENSG00000145335). Some of **t**hese are tissue-specific. The largest isoform group, which consist of 140 aa, was shown to accumulate in dopaminergic neurons of PD patients as Lewy bodies or Lewy neurites [18–20,43,44]. The luciferase-/or GFP-puromycin marker was inserted between the last coding codon GCC and the stop codon TAA (GCC/TAA) in exon 6 of the *SNCA* gene.

### Creation of SH-SY5Y cell lines expressing α-synuclein-GFP

**Fig 2:**
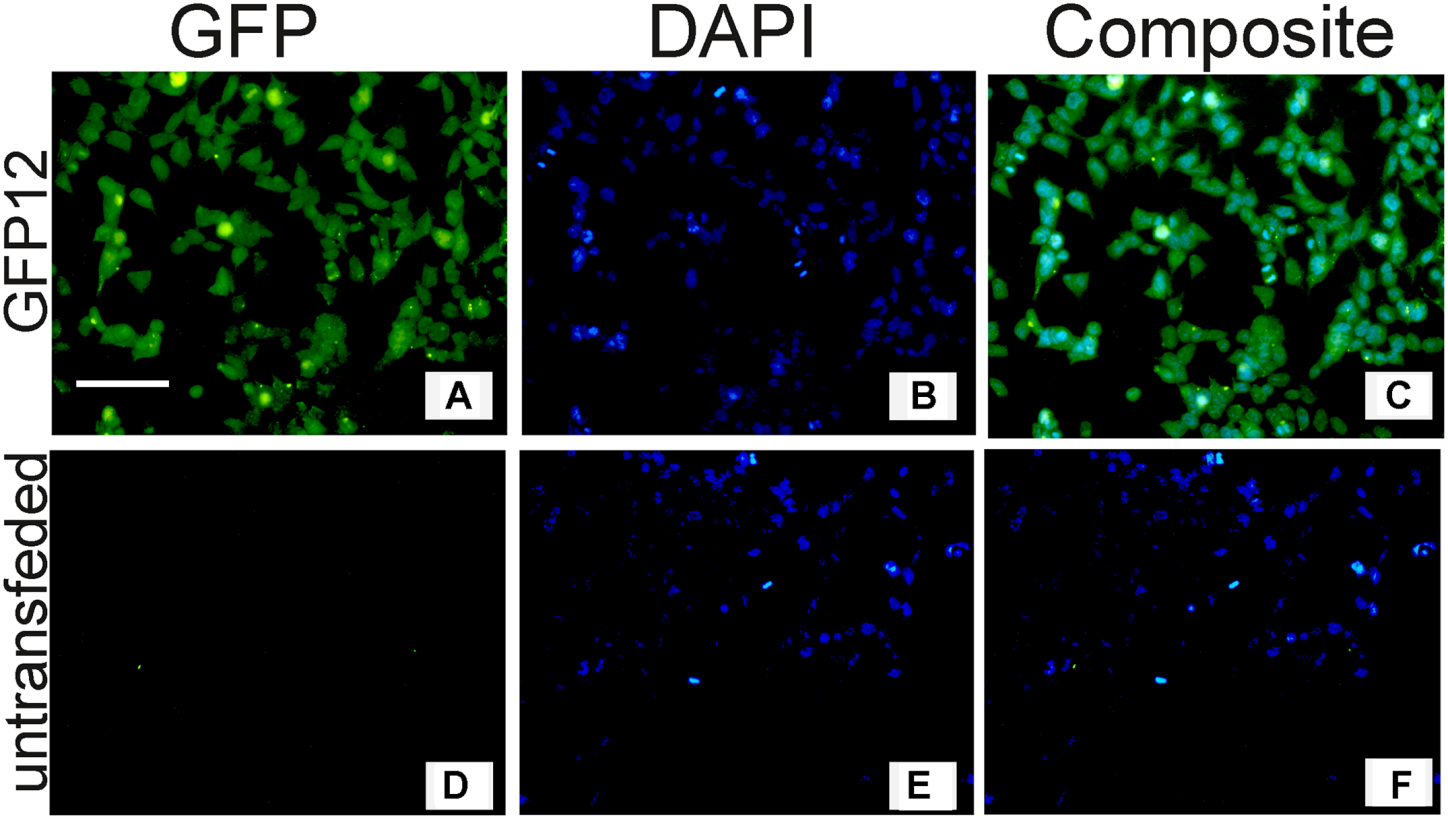
Fluorescent Imaging of GFP and DAPI in GFP12 (A-C) and untransfected SH-SY5Y (D-F) cells. Cells were grown for 48 hr, stained with DAPI, and visualized by immunofluorescent microscopy. All selected cells expressed GFP suggesting that the GFP12 cell line was efficiently selected by 10 μg/ml puromycin. Bar = 30 μm.

To eliminate the production of free GFP or luciferase, we generated donor plasmids containing no ATG initiation on the reporter gene, the *SNCA-GFP-2A-Puro* and *SNCA-luc-2A-Puro* donor plasmids (Fig 1). The *SNCA-GFP-2A-Puro* or *SNCA-luc-2A-Puro* donor plasmid was cotransfected with the right and left Zinc Finger Nuclease (ZFN) plasmids (Fig 1) into SH-SY5Y cells. Transfected SH-SY5Y cells were transferred to 6-well dishes, and selected with 10 μg/ml puromycin. After 1–2 weeks of puromycin selection, a mixed population of colonies was formed. The colonies in each well were pooled and recultured for characterization and storage. Using these approaches, we created one SH-SY5Y cell line that expressed α-syn-GFP; we labeled this cell line GFP12 (Fig 2, S2 Fig) and two cell lines that expressed α-syn-luc fusion protein, we labeled these cell lines Luc6B and Luc6B-5. Since the Luc6B-5 expressed a lower amount of α-syn-luc mRNA than the Luc6B (S3 Fig), the Luc6B cell line was selected for detailed analyses.

To confirm whether the GFP12 cell line expressed α-syn-GFP, cells were plated in 6-well plates, and fluorescent images were taken 48 hours later. Fig 2 shows that all puromycin-selected cells were GFP positive suggesting that 100% of the GFP12 cells expressed GFP (Fig 2). Additionally, immunofluorescent staining with α-syn antibody of GFP12 cells showed colocalization of α-synuclein and GFP in GFP expressing cells (GFP12) confirming that GFP-positive cells expressed α-syn-GFP fusion protein (S2 Fig). The lack of colocalization between endogenous α-synuclein and α-syn-GFP in some of the GFP positive cells observed in S2 Fig was expected since the GFP12 cell line consists of a mixture of transfected cells.

**Fig 3:**
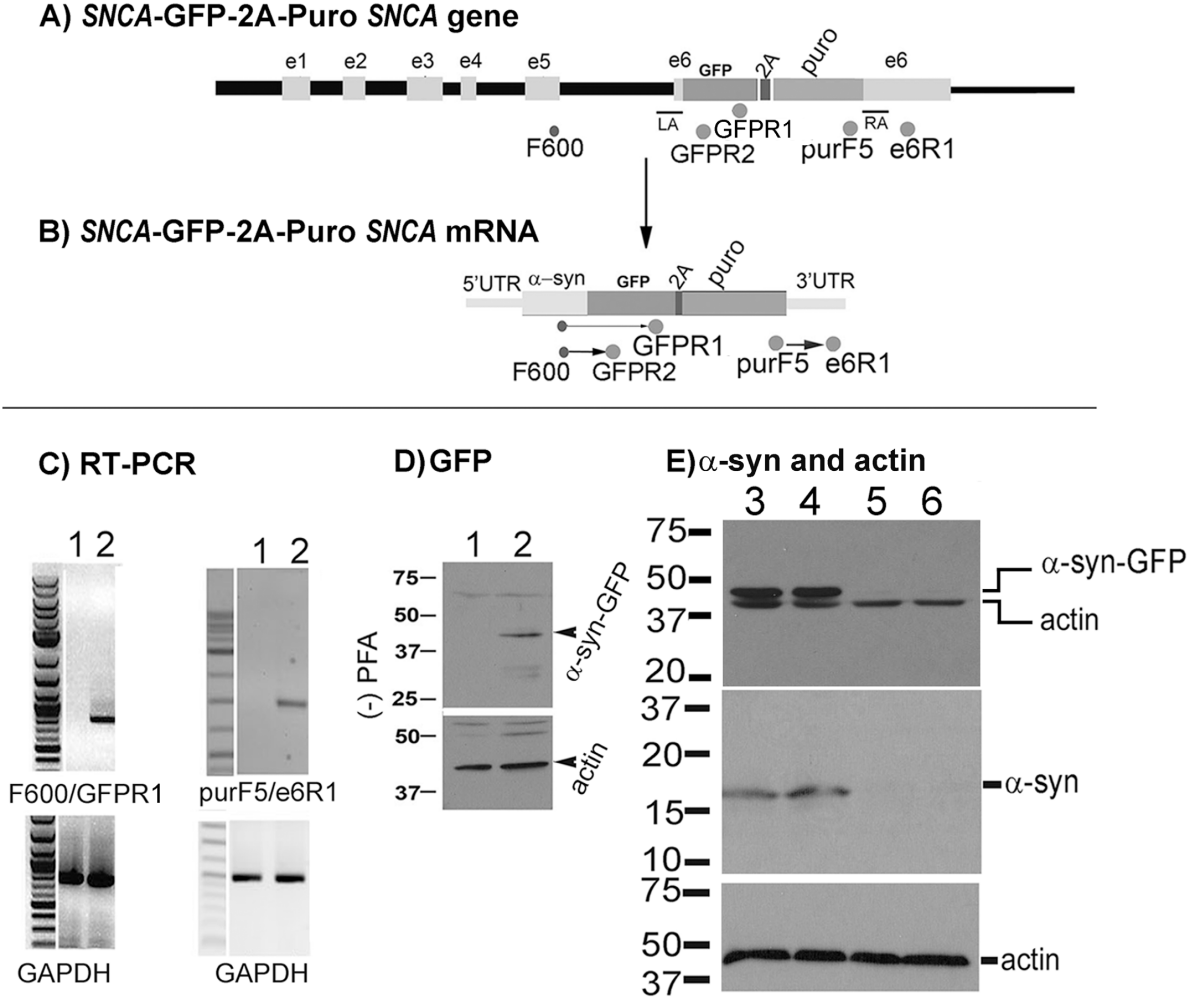
Expression of α-syn-GFP by the GFP12 cell line. **(A)** and **(B)** schematic representation of the modified *SNCA* gene and mRNA containing the *GFP-2A-Puro* gene cassette. **(C)** RT-PCR products of the GFP12 mRNA using the F600/GFPR1 (1 kb), purF5/e6R1 (1.5 kb), and GAPDH primer pairs. Both primer pairs detected the expected *SNCA* mRNA products in the GFP12 (lane 2) but not in SH-SY5Y control (lane 1) cells suggesting that the *GFP-2A-Puro* cassette was inserted into the correct locus. **(D)** and **(E)** Western blots of the GFP12 cell line using antibodies to GFP (**D**), α-syn (**E**), and actin. Both GFP and α-syn antibodies detected a band at about 45 kDa, corresponding to the predicted MW of the α-syn-GFP fusion protein (lanes 2, 3, 4) that was not seen in SH-SY5Y cells (lane 1). To improve detection of wild type α-synuclein, the immunoblot was first incubated with 0.4% PFA for 30 min prior to incubation with the primary antibody. **(E)** Western blots of GFP12 transfected with 100 nM control siRNA control (lanes 3, 4) and 100 nM *SNCA* siRNA (lanes 5, 6) and detected with α-synuclein antibody 211 (Santa Cruz Biotech). GFP12 cells were transfected with 100 nM control siRNA and *SNCA* siRNA for 5 days. The *SNCA* siRNA inhibited the expression of both the α-syn-GFP fusion protein and wild type α-synuclein but not actin (lanes 5, 6).

RT-PCR analysis using the F600/GFP-R1 and purF5/e6R1 primer pairs of RNA samples from the GFP12 cell line produced the expected PCR products for each primer pair (Fig 3C). The F600/GFPR2 primer pair is located at the α-syn mRNA/ GFP junction, while the purF5/e6R1 primer pair is located at the puromycin/3’-UTR α-syn mRNA junction. The positive RT-PCR bands showed that the *GFP-2A-Puro* reporter gene cassette was properly inserted into the *SNCA* locus.

Western blots using protein extracts from the GFP12 line and antibodies to GFP and α-synuclein confirmed that the donor plasmid generated only the α-syn-GFP fusion protein at about 45 kDa, which corresponded to the calculated MW of the α-syn-GFP fusion protein (α-syn MW = 18–20 kDa, GFP = 27 kDa). No additional bands corresponding to free GFP were detected (Fig 3D, lane 2). The α-syn antibody (211, Santa Cruz) detected an α-syn-GFP band at 45 kDa (Fig 3E, lanes 3–4, top) and the wild type band at 18 kDa (Fig 3E, lanes 3–4, middle) in the control siRNA transfected GFP12 cells. Both the endogenous α-synuclein and α-syn-GFP fusion proteins were depleted in GFP12 cells transfected with α-syn siRNA but not the actin band (Fig 3E, lanes 5–6). When using the α-syn antibodies (Santa Cruz, cat # sc-12767, 211 or cat# sc-58480, designated LB509), blots were cross-linked by preincubating with 0.4% PFA for 30 min [42] to improve detection of the endogenous α-synuclein.

### Generation of SH-SY5Y cell lines expressing full-length α-synuclein-luciferase (α-syn-luc) fusion proteins

**Fig 4:**
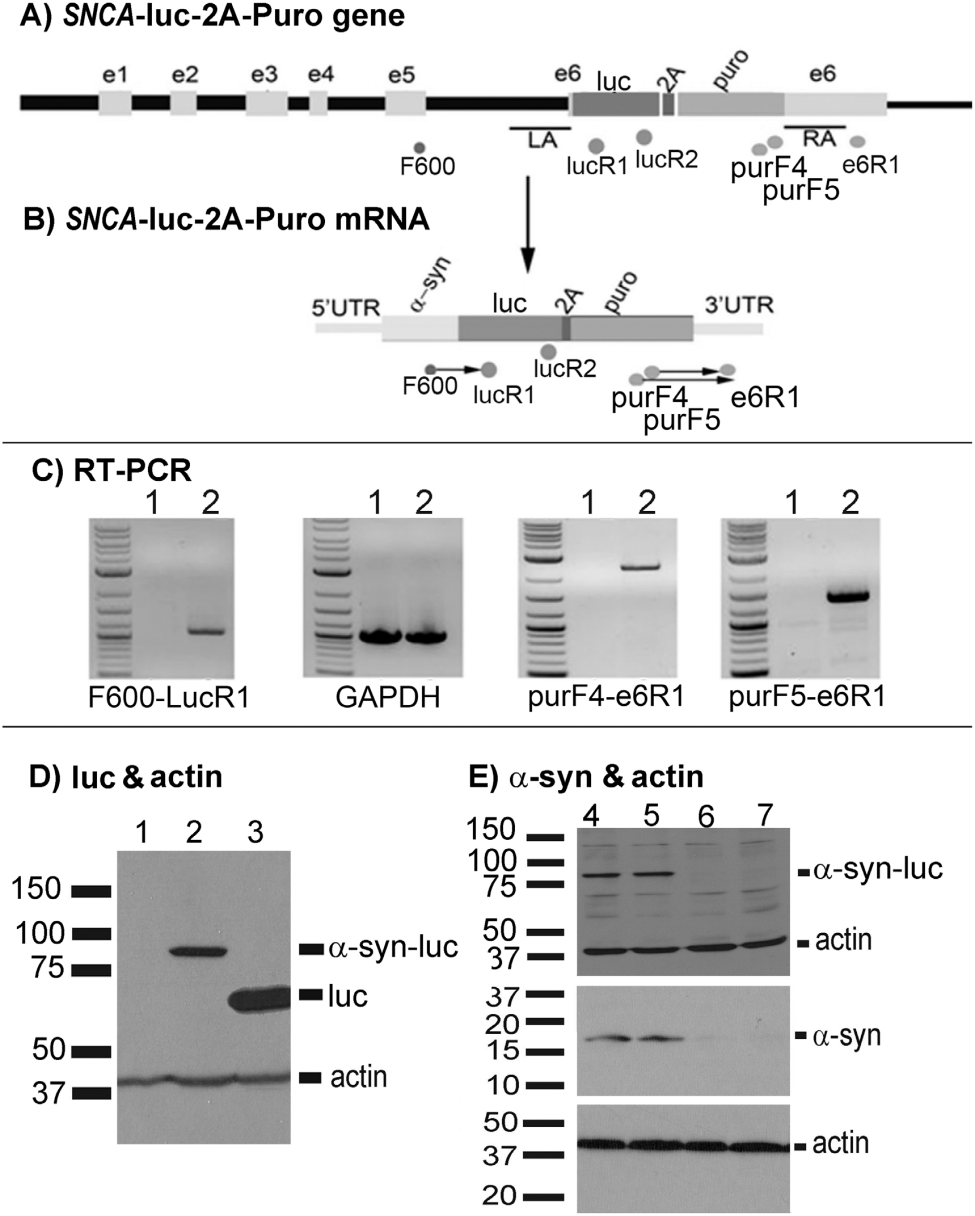
Characterization of the Luc6B cell line. Luc6B cells were grown in DMEM/FBS/puromycin medium for 48 hours, RNA and proteins were extract for RT-PCR, qPCR, and Western blots. **(A)** and **(B)** schematic representation of the modified *SNCA* gene and mRNA containing the *luc-2A-Puro* gene cassette. **(C)** RT-PCR of the Luc6B cells using the F600/LucR1, purF4/e6R1, and purF5/e6R1 and GAPDH primer pairs. All three primer pairs produced the expected bands in the luc6B (lane 2) but not in SH-SY5Y (lane 1) control cells suggesting that the *luc-2A-Puro* gene cassette was inserted into the proper targeted locus. **(D)** Western blots of SH-SY5Y (lane 1), Luc6B (lane 2), and pGL2-CMV-luc transfected SH-SY5Y cell extracts using antibodies to luciferase and actin. The luciferase antibody detected a band at about 77–79 kDa in Luc6B cells (lane 2) and a 60 kDa band in pGL2-CMV-luc transfected cells (lane 3) but not in untransfected SH-SY5Y cells (lane 1). **(E)** Western blots of cell extracts from Luc6B cells transfected with 100 nM of control siRNA (lanes 4, 5) and *SNCA* siRNA (lanes 6, 7) using α-syn antibody 211 and actin. The 211 antibody detected the wild type α-synuclein and the luciferase tagged α-synuclein (77–79 kDa) in the control siRNA transfected Luc6B cells but not in the *SNCA* siRNA transfected Luc6B cells. The *SNCA* siRNA transfection did not have any effect on actin expression (lanes 6, 7).

Transfection of SH-SY5Y cells with *ZFN-FokI* plasmid and the *SNCA-Luc-2A-Puro* donor plasmid resulted in two α-syn-luc expressing cell lines, designated Luc6B and Luc6B-5. We selected the Luc6B cell line for detailed analyses since the Luc6B-5 cell line expressed a lower amount of α-syn-luc mRNA (S3). Fig 4 describes the results of RT-PCRs and Western blot analyses of the Luc6B line. RT-PCRs using primer pairs F600/LucR1 and purF4/e6R1, and purF5/e6R1 (Fig 4B) generated RT-PCR products that were absent from untransfected cells. The sizes of these PCR products were consistent with the predicted PCR fragment for these primer pairs (Fig 4C). These results confirmed that the *luc-2A-Puro* cassette was inserted correctly into the targeted *SNCA* locus. RT-PCR using the control *GAPDH* primer pair showed that RNA samples extracted from the Luc6B and untransfected SH-SY5Y cells were of high quality and quantitatively equal.

Luciferase antibody detected a band of about 77 kDa predicted for the α-syn-luc fusion protein in protein extracts from the Luc6B cell line (Fig 4D, lane 2) but only a 60 kDa band from SH-SY5Y cells transfected with the pGL2-CMV-luc positive control plasmid (Fig 4D, lane 3). No additional high MW band was detected in the untransfected SH-SY5Y cells (Fig 4D, lane 1). The α-synuclein antibody (211) detected the endogenous 18 and 77 kDa bands, predicted sizes of untagged α-synuclein and α-syn-luc, respectively, from lysates of Luc6B cells transfected with a control siRNA (Fig 4E, lanes 4 and 5). The observation of both the untagged α-synuclein (α-syn) and tagged α-syn-luc proteins indicated that only one copy of the *SNCA* locus was modified by ZFN. The α-syn 211 antibody failed to detect both the endogenous 18 and 77 kDa α-synuclein bands in protein extracts from Luc6B cells transfected with 100 nM of α-syn siRNA (Fig 4E, lanes 6–7). These observations confirm that the Luc6B cell line specifically expressed the α-syn-luc fusion protein.

### Effects of valproic acid on Luc6B and GFP12 expression of α-synuclein

VPA is a histone deacetylase (HDAC) inhibitor that modifies gene expression via epigenetic mechanisms [45,46]. HDAC inhibitors modify gene expression by increasing the level of histone acetylation, generally resulting in increased gene expression. VPA increased the levels of endogenous α-synuclein in SH-SY5Y and was found to be neuroprotective against rotenone cytotoxicity [40,46] and mouse cerebral neuron-enriched cultures [45]. VPA-induced increase of α-synuclein was accompanied by increased protection against glutamate-induced neurotoxicity [47]. We hypothesized that VPA treatment of GFP12 and Luc6B cells would increase α-syn-GFP and α-syn-luc expression, while also realizing that VPA treatment does not necessarily increase the expression of all genes.

**Fig 5:**
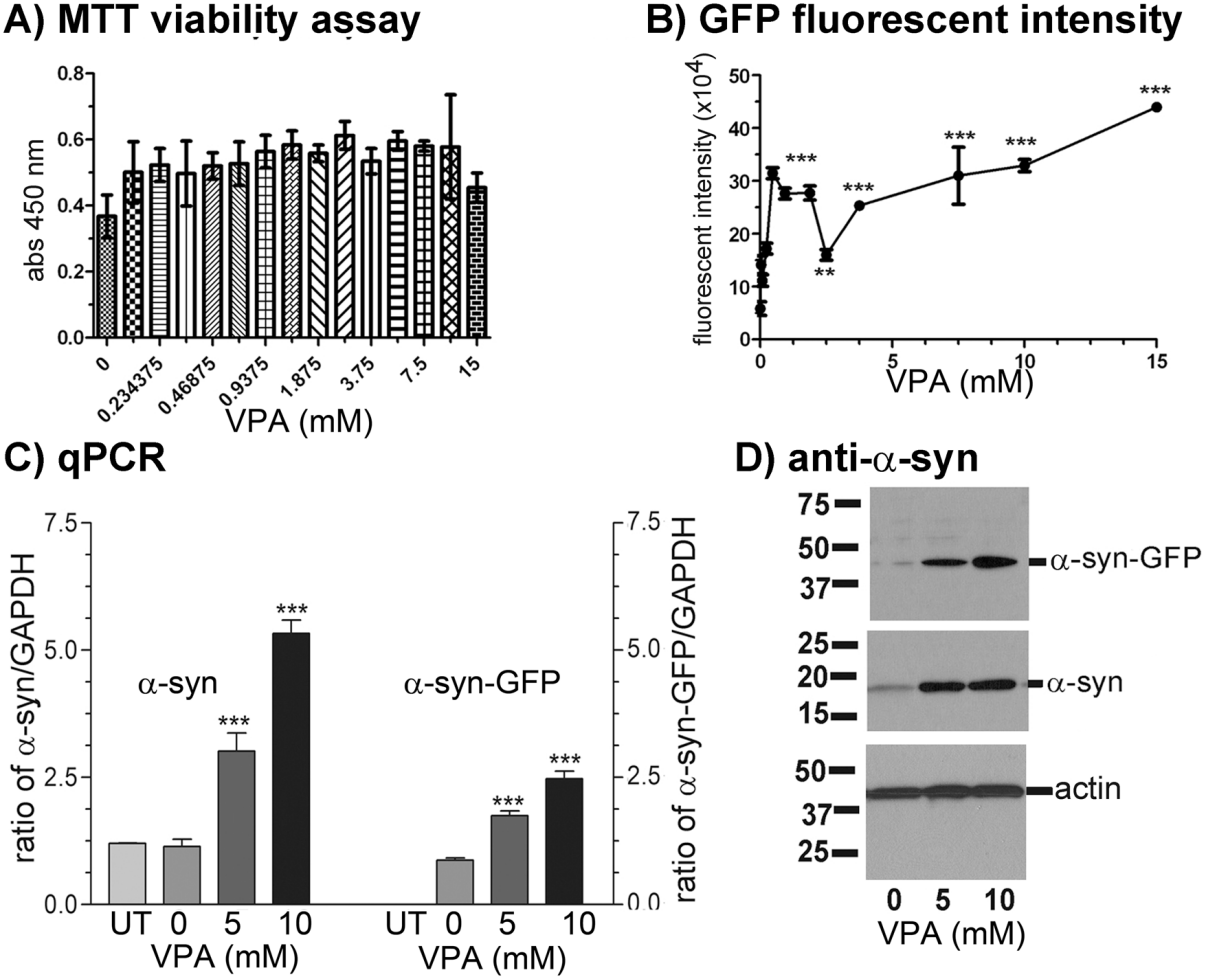
Effect of valproic acid (VPA) treatment on α-syn-GFP fusion protein expression in GFP12 cells. GFP12 cells were grown in 384-well plates with VPA in DMEM/FBS medium. GFP intensity was measured using a DTX880 plate reader with excitation wavelength set at 485 nm and emission wavelength set at 535 nm, followed by MTT viability assay. **(A)** MTT viability assay shows that GFP12 cell growth was not significantly affected by increasing VPA concentration. **(B)** Effect of VPA on GFP12 fluorescent intensity. Fluorescent intensity of GFP12 cells increased with higher concentrations of VPA. Data were analyzed by One-way ANOVA (P <0.0001, n = 5) followed by Tukey’s multiple comparison test. Values were expressed as Mean ± SD. **(C)** qPCR and **(D)** Western blots of VPA treated GFP12 cells. qPCR shows significant increase of both wild type (α-syn) and GFP tagged α-synuclein (α-syn-GFP) mRNA. Similarly, Western blots using α-syn antibody 211 show that VPA treatment caused a significant dosage-dependent increase in the expression of both wild type α-synuclein and α-syn-GFP fusion protein by GFP12 cells. * P < 0.05, ** P < 0.01, *** P < 0.001.

**Fig 6:**
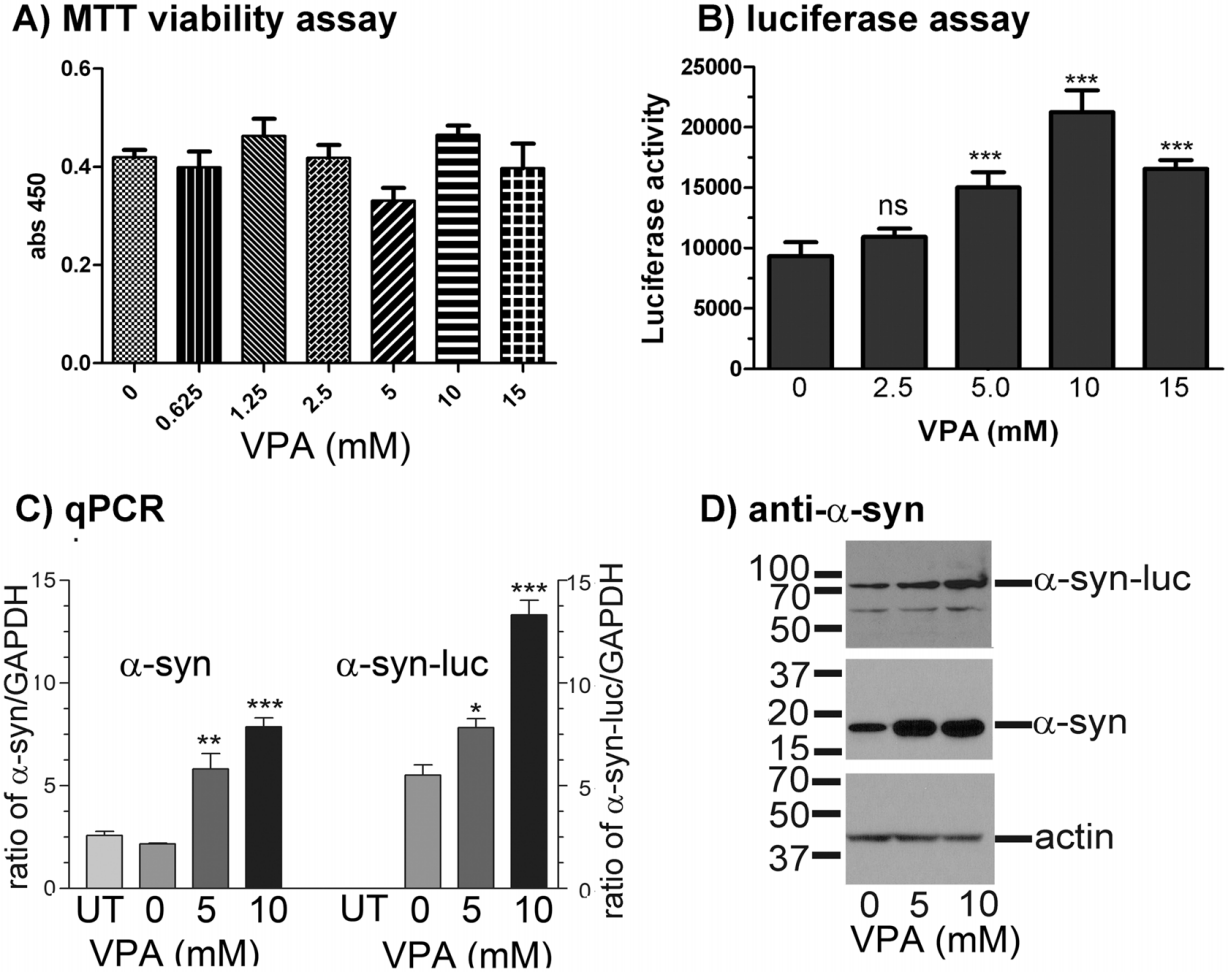
Effects of VPA treatment on α-syn-luc expression in the Luc6B cell line. **(A)** MTT viability assay of Luc6B cells (n = 4). Cells were plated in 384-well tissue culture plate in DMEM/FBS containing the indicated VPA concentration, and grown for 72 hours. Luc6B cell growth was not significantly affected by VPA concentration. **(B)** Luciferase assay of VPA treated cells. n = 4. Luciferase activities increased with increasing concentrations of VPA in Luc6B cells. Luciferase activity was expressed as Mean ± SD. **(C)** qPCR and **(D)** Western blots of mRNA and protein extracted from VPA-treated Luc6B cells. The ratio of both α-syn/GAPDH and α-syn-luc/GAPDH mRNA increased significantly with increased concentrations of VPA when comparing with mRNA expression of untreated Luc6B cells. UT = untransfected SH-SY5Y cells. Data for luciferase enzymatic activity and qPCR were analyzed by one-way ANOVA, P<0.0001 followed by Tukey’s multiple comparison test. The P values represent the difference between untreated and VPA treated Luc6B cells. * P < 0.05, ** P < 0.01, *** P < 0.001.

VPA treatments for 72 hours did not affect the viability of the GFP12 (Fig 5A) and Luc6B cells (Fig 6A) significantly. VPA treatments increased α-syn-GFP signals and α-syn-luciferase activities in a dose-wise manner in GFP12 (Fig 5B) and Luc6B (Fig 6B) cell lines, respectively. VPA caused an incremental increase of α-syn-GFP significantly starting at 0.234 mM. The fluorescent intensity increased with increasing concentrations of VPA. Statistical analysis was done by One-way ANOVA P<0.0001 followed by Tukey-Kramer Multiple Comparison Test. Similar to the GFP12 cell line, VPA treatment caused a significant increase of α-syn-luc activities in Luc6B cells (One-way ANOVA, P<0.0001, Tukey Multiple Comparison Test, P<0.001, n = 4). The mean and SD luciferase activities for the VPA treated Luc6B cells were 9,337±1,158, 10,928±685, 15,030±1,259, 21,266±1,796, and 16,577±697 at 0, 2.5, 5, 10, and 15 mM VPA, respectively.

Both GFP12 and Luc6B cell lines were further validated by qPCR of RNA (Figs 5C and 6C) and Western blots of protein extracts (Figs 5D and 6D) from VPA treated cells. VPA treatments caused a significant increase of endogenous α-synuclein mRNA in GFP12 (Fig 5C, left) and Luc6B (Fig 6C, left) when the primer pair, F600/*SNCA*-R, was used for qPCR. The endogenous α-synuclein was absent when primer pair specific for α-syn-GFP mRNA (F600/GFP-R2; Fig 5C, right) or α-syn-luc mRNA (F600/LucR2; Fig 6C, right) was used for qPCR. Compared with untreated GFP12 or Luc6B cells, the levels of endogenous α-synuclein and fusion proteins (α-syn-GFP and α-syn-luc) at 10 mM of VPA increased 2.5 to 3 folds. Similarly, VPA treatments also increased the amounts of α-syn-GFP (Fig 5D) and α-syn-luc (Fig 6D) fusion proteins as shown by Western blots using the α-syn antibody 211.

### Quality of luciferase signal from the Luc6B cell line

**Fig 7:**
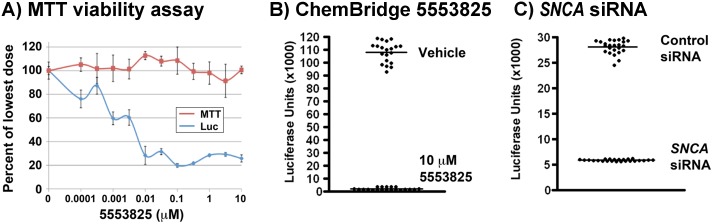
High throughput screening quality metrics for the Luc6B cell line. **A&B)** Quality metrics for Luc6B treated with luciferase inhibitor ChemBridge 5553825 vs. DMSO. **(A)** We identified ChemBridge 5553825 as a potent *ATXN2-luc* inhibitor reducing expression by 80% at 0.1 nM (blue) that does not inhibit cell growth at any tested dose (red, MTT assay). Tests were conducted using SH-SY5Y cells stably expressing *ATXN2-luc*, with treatment time of 48 hours. We ultimately determined that ChemBridge 5553825 is a luciferase inhibitor. **(B)** 25,000 Luc6B cells per well were exposed to 10 nM compound in 50 μl total volume in a 384 well plate (n = 20 per condition). Luciferase expression was determined after 24 hrs. Shown is a scatter plot with means indicated. Mean and SD for 5553825 and 1% DMSO vehicle were 2023±1030 and 108,252±7929, respectively. Using these values, the computed quality metrics for Luc6B when treated with luciferase inhibitor are as follows: *Z*’-score: 0.75, Signal/Noise (*S/N*): 15.4, Signal/Background (*S/B*): 53, Coefficient of variation (*C*_*v*_): 6.5%. **(C)** Quality metrics for Luc6B cells treated with *SNCA* siRNA vs. control siRNA. Luc6B cells were transfected with *SNCA* siRNA or control siRNA for 5 days, then trypsinized and distributed at 30,000 cells/well in 24 wells of a 384 well plate, cultured for an additional 24 hours, after which time luciferase assays were performed. Shown is a scatter plot with means indicated. The Mean and SD for *SNCA*-siRNA and control siRNA were 5,886±163 and 28,093±1,344, respectively. Using these values, the computed quality metrics for Luc6B when treated with *SNCA*-siRNA are as follows: *Z*’-score: 0.80, *S/N*: 18.8, *S/B*: 4.8, *C*_*v*_: 5.3%. Computations were made as follows: *Z*’-score = 1-[(3(σ_exp_+σ_cont_))/|μ_exp_-μ_cont_|]; *S/N* = |μ_exp_-μ_cont_|/|σ_exp_-σ_cont_|, *S/B* = μ_cont_/μ_exp_; *C*_*v*_ = 1/*S/N*, where μ and σ are the mean and SD, respectively.

We determined the quality metrics of high-throughput screening (HTS) for the Luc6B cells line using luciferase inhibitor ChemBridge 5553825 and *SNCA* siRNA. The luciferase inhibitor ChemBridge 5553825 was determined previously as a nontoxic compound potently inhibiting luciferase (Fig 7A). After 24 hr treatment, the means and SDs for 10 μM and 1% DMSO vehicle were 2023±1030 and 108,252±7929, respectively (Fig 7B). Following Zang et al. [48], we computed the *Z*’-score = 0.75 with Signal/Noise (*S/N*) = 15.4, Signal/Background (*S/B*) = 53, and Coefficient of variation (*C*_v)_ = 6.5%). To calculate the quality metrics of the Luc6B cell line in response to *SNCA* siRNA expression inhibition, Luc6B cells transfected with control siRNA and *SNCA* siRNA for 5 days were transferred to 384-well plate (n = 24), and luciferase activity was measured 24 hours later. The means and SD for *SNCA* siRNA and control siRNA were 5,886±163 and 28,093±1,344, respectively. Using these values, the computed quality metrics for the Luc6B cell line, when treated with *SNCA* siRNA, were as follows: *Z*’-score: 0.80, *S/N*:18.8, *S/B*: 4.8, *C*_*v*_: 5.3% (Fig 7C). The high Z’-scores calculated from inhibiting luciferase activity by ChemBridge 5553825 (Z‘-score: 0.75) and *SNCA* siRNA (Z‘-score: 0.80) support the use of the Luc6B cell line in HTS.

## Discussion

Parkinson’s Disease (PD) is caused by the loss of dopaminergic neurons in the substantia nigra. Approximately 15 pathogenic mutated genes are associated with familial and sporadic PD [3]. Among these PD associated genes are 7 genes that are linked to autosomal dominant PD. One of these is *SNCA*, encoding α-synuclein (α-syn). To date, 5 rare missense mutations have been found in *SNCA*, including A53T [4], A30P [7], E46K [7], H50Q [1], and G51D [8]. In addition to missense mutations, duplication [2] and triplication of the *SNCA* gene [5,6,49] have been found in both autosomal dominant and sporadic PD [3,5]. Overexpression of α-synuclein caused neurotoxicity in cell, mouse, rat, *C*. *elegans*, and *Drosophila* models [3,10,12–14,23]. Likewise, brain tissues from PD patients also showed high levels of α-synuclein mRNA [18,19,43] and protein [32,49]. Together, these observations suggest that elevated levels of α-synuclein cause the death of dopaminergic neurons in Parkinson’s Diseases. Consistent with this, reducing the intracellular levels of wild type α-synuclein, alleviated neurotoxicity and reduced Lewy body pathology [23,24,50–52]. Toward finding compounds that reduced the levels of excess α-synuclein, we produced cell lines expressing either α-syn-GFP or α-syn-luc using the ZFN genome editing technique. The resulting cell lines expressed full-length α-syn-GFP or α-syn-luciferase fusion proteins under the control of the intact endogenous system of expression regulation.

Expression of α-synuclein is regulated at multiple points such as the transcriptional, translational, and degradation levels. At the transcriptional level, *SNCA* expression is regulated by the 400 bp promoter/enhancer domain, *NACP-REP1* [27,29,32,33], located at about 8852 bp upstream of the transcription start site [28] which is flanked by two enhancer domains. Standard generation of cell lines using a transfected plasmid with short promoters would miss compounds acting on distant regulatory domains. The GFP12 and Luc6B cell lines, generated by directed insertion of the reporter gene (*GFP* or *luciferase*) in-frame with the *SNCA* gene, has none of the above disadvantages since the tagged α-synuclein expression is under the control of the entire transcription regulatory system. This property will allow for the discovery of compounds that affect all possible regulatory domains/elements of the expression of the *SNCA* gene.

Chromatin structure influences the overall expression of the transcriptome. Valproic acid is one chromatin remodeling inhibitor that we chose to evaluate using our cell models since it had previously been demonstrated to be neuroprotective in cell and animal models of PD [40,45–47]. Increased levels of both *SNCA* mRNA (Figs 5C and 6C) and tagged α-synuclein (α-syn-GFP and α-syn-luc) (Figs 5D and 6D) upon treatment with VPA suggested that altered chromatin structure in our cell line model is associated with *SNCA* activation. The non-specific nature of VPA to act genome-wide is likely why neuroprotective properties for VPA have been observed in PD models despite its known function to increase *SNCA* expression [40,45–47].

α-Synuclein clearance occurs through either lysosomal or ubiquitin-dependent proteasomal pathways [53–58]. Inhibiting either of these two pathways would increase the intracellular levels of α-synuclein. Luc6B cell lines treated with bafilomycin A1 for 24 hours increased luciferase activity (S4 Fig) indicating that the level of α-syn-luc fusion protein had increased. This observation was in line with previous observations that bafilomycin A1 treatment increased α-synuclein [54,57].

RNA binding proteins, microRNAs, and short noncoding RNAs, which interact with either 3’ or 5’ UTRs, may also regulate the expression of α-synuclein. One such study included a screen of >300,000 small molecules inhibiting *SNCA* expression, and identified compounds that interfered with iron-regulatory protein 1 (IRP-1) binding to the *SNCA* 5’ stem-loop altering α-synuclein expression in H4 and SH-SY5Y neuroblastoma cells [59,60]. The disadvantage of the study was that the screening assay included only the *SNCA* 5’-UTR, eliminating the ability to identify inhibitors acting at other *SNCA* regions. Our GFP12 and Luc6B cell lines offer the advantage of a complete, unmodified 5’ UTR and 3’ UTR sequences, which allow for the discovery of compounds acting at these *SNCA* regions to modify *SNCA* expression or mRNA stability.

Clearance of wild type and accumulated α-synuclein occurs through the lysosomal, endosomal and ubiquitin mediated proteasomal pathways [53,61–63]. The ubiquitin-proteasomal pathway [64] mostly mediates wild type α-synuclein clearance, while clearance of aggregated, misfolded, or accumulated α-synuclein occurs through the autophagy dependent lysosomal pathway [65,66]. Discovery of compounds that can enhance the function of these pathways will reduce the accumulation of α-synuclein levels in the cell. For example, compounds that enhance the enzymatic activity or increase the expression of ATP13A2 enzyme, a protein associated with Parkinson’s disease 9 (PARK9), would increase the clearance of accumulated α-synuclein via the lysosomal pathways [67–70]. Alternatively, compounds that enhanced the ubiquitin-dependent clearance of misfolded E3 ubiquitin ligase Nedd4, or increased Nedd4 expression protected against α-synuclein induced toxicity in Drosophila and reduced α-synuclein accumulation leading to decreased DA cell death in the rat substantia nigra [71].

As accumulation of wild type and mutant α-synuclein is pathogenic in PD and a feature of familial PD and other α-synucleinopathies, compounds that reduce the level of α-synuclein may alleviate and prevent the occurrence of Parkinsonism phenotypes. The Luc6B and GFP12 cell lines offer many advantages over other cell lines expressing only partial *SNCA* mRNA or partial promoter/enhancer sequences for HTS to discover compounds that reduce the level of α-synuclein. These advantages include the preservation of the intact transcriptional regulatory system, the 5’ UTR, and 3’ UTR. Therefore, *SNCA* expression in the Luc6B and GFP12 cell lines is under the control of the complete transcriptional regulatory system. This allows the detection of compounds that specifically affect important *SNCA* transcriptional domains/elements or compounds that have global effects on the transcription of the *SNCA* gene that would be missed if cell line used expresses only a partial regulatory DNA segment. Furthermore, the presence of unmodified structures of the 5’UTR and 3’UTR of *SNCA-Luc* or *SNCA-GFP* hybrid mRNA allow for discovery of compounds that interfere with regulatory proteins that interact with these two UTR regions.

Additional advantages include the expression of full-length α-synuclein fused with luciferase or GFP, allowing for the discovery of compounds that affect all clearance pathways, be they lysosomal, proteasomal, or endosomal pathways such as iron-responsive protein 1 (IRP-1) or microRNAs [47]. Furthermore, the α-syn-luc or α-syn-GFP fusion protein produced by the Luc6B or GFP12 cell line allows for fine evaluation of *SNCA* expression with varying compound doses [72].

## Conclusions

The high *Z*’-score of luciferase activity supports the use of the Luc6B cell line for HTS (Fig 7). Furthermore, the GFP12 cell line can be used as an independent cell line assay for validation of compounds, and is amenable to high-content assays and evaluation of compound effects on α-synuclein aggregation and subcellular localization.

The cell lines described in this study represent a unique resource for high-throughput compound screening to discover drugs that reduce cellular α-synuclein protein levels. Finally, the successful generation of the Luc6B and GFP12 cell lines demonstrate that ZFN-mediated knock-in of a reporter gene into a designated gene locus is a valuable tool for generating cell lines for HTS.

## Supporting Information

S1 Fig Cel-1 assay analysis of the ZFN and the identification of the cleavage site and ZFN binding domains of ZFN on the *SNCA* DNA (Sigma Aldrich). This assay was done by Sigma Aldrich to confirm the ZFN specificity. **(A)** DNA and RNA from untransfected and transfected cells were transfected with the left and right ZFN plasmids and grown for 2 days to allow random DNA repair. DNA and RNA samples were isolated. PCR was performed using a primer pair across the targeted cleavage site, and the PCR product, 329 bp, was treated with CEL-1, an endonuclease isolated from celery. CEL-1 has high specificity for mismatches, insertions, and deletions in DNA.CEL-1 mediated cleaved of the ZFN mutated PCR fragment generated two bands of 195 and 134 bp from the 329 bp fragment. **(B)** Cleavage site of the ZFNs located 59 bp from the TAA stop codon of the *SNCA* gene.
(TIF)

S2 Fig Immunofluorescent staining of the GFP12 cell line. **(A)** GFP, **(B)** α-syn antibody, **(C)** overlay of GFP and anti-α-syn staining, **(D)** overlay of anti-GFP, anti-α-syn, and DAPI. The non-uniformity between GFP and α-synuclein labeling exists since the GFP12 cell line contains a mixed population of transfected cells.
(TIF)

S3 Fig RT-PCR of Luc6B and Luc6B-5 cell lines. RT-PCR amplicons of RNAs isolated from Luc6B and Luc6B-5 cells using the F600/lucR1, F600/lucR2 and GAPDH primer pairs. Both primer pairs, F600/lucR1 and F600/lucR2, produced the correct bands at the predicted size for fragments generated by these primer pairs. Lane 1, SH-SY5Y, lane 2, Luc6B-5, and lane 3, Luc6B. These results showed that Luc6B cells expressed a high level of α-syn-luc mRNA than the Luc6B-5 cell line. Therefore, the Luc6B cell line was selected for detailed studies.
(TIF)

S4 Fig Effects of bafilomycin A1 on the Luc6B cell line. Bafilomycin A1 treatment increased the level of luciferase activities in Luc6B cells. SH-SY5Y (UT). and Luc6B cells were cultured in 6-well dishes, and grown in DMEM/FBS medium containing 50 μM retinoic acid for 8 days to differentiate cells into neuron-like cells. Cells were transferred to clean wells every 3–4 days. On the day prior to the experiment, cells were transferred to clean wells. The next day, cells were treated with DMSO, 20 nM, and 200 nM of bafilomycin A1. Luciferase activity was measured 24 hrs later using Promega Luciferase detection kit. Bafilomycin A1 was purchased from Sigma Aldrich.
(TIF)

S1 Table Oligonucleotide sequences of PCR primers used in this manuscript for RT-PCR or qPCR. (XLSX)
