# Correction: Generation of *SNCA* Cell Models Using Zinc Finger Nuclease (ZFN) Technology for Efficient High-Throughput Drug Screening

**DOI:** 10.1371/journal.pone.0256366

**Published:** 2021-08-12

**Authors:** Warunee Dansithong, Sharan Paul, Daniel R. Scoles, Stefan M. Pulst, Duong P. Huynh

The cell lines used in this study [1] were recently subjected to cell line authentication in which the GenePrint 24 system (Promega) was used to conduct short tandem repeat (STR) analysis on 24 loci. This analysis revealed that the cells referred to in the article as SH-SY5Y or as derivatives of SH-SY5Y cells (including Luc6B and GFP lines), were instead HEK-293. The authors apologize that this issue was not identified prior to the article’s publication.

The intention of this study was to validate cell line assays for high-throughput screening to identify compounds which lower *SNCA* expression. The determination that the cells are HEK-293 rather than SH-SY5Y in origin does not impact the validity of the system or results reported in the article, including the validation of the reported assay and cell lines for identifying compounds that modulate *SNCA* expression. Nevertheless, readers should consider potential implications of the HEK-293 cellular context when using this system in future studies.

A reviewer raised that HEK-293 cells are not expected to differentiate in response to retinoic acid as is reported in the S4 Fig legend. This figure showed that autophagy blockade increased α-synuclein levels. The authors stand by the validity of this result which has since been confirmed in other studies (e.g. [2, 3]), although the updated information about the cell line calls into question details in the figure legend about differentiation of the cells into neuron-like cells in response to retinoic acid.

The underlying data to support all graphs reported in the article are available upon request from DRS.

DPH is deceased, and so as of the time of this notice, DRS will serve as this article’s corresponding author: Department of Neurology, University of Utah, 175 North Medical Center Drive East, 5th Floor, Salt Lake City, Utah, 84132, United States of America. Daniel.Scoles@hsc.utah.edu.
