# Supplementary figures and images for: Generation of SNCA Cell Models Using Zinc Finger Nuclease (ZFN) Technology for Efficient High-Throughput Drug Screening
Source: PLoS One. 2015 Aug 28;10(8):e0136930. doi: 10.1371/journal.pone.0136930 (PMC4552753; doi:10.1371/journal.pone.0136930)

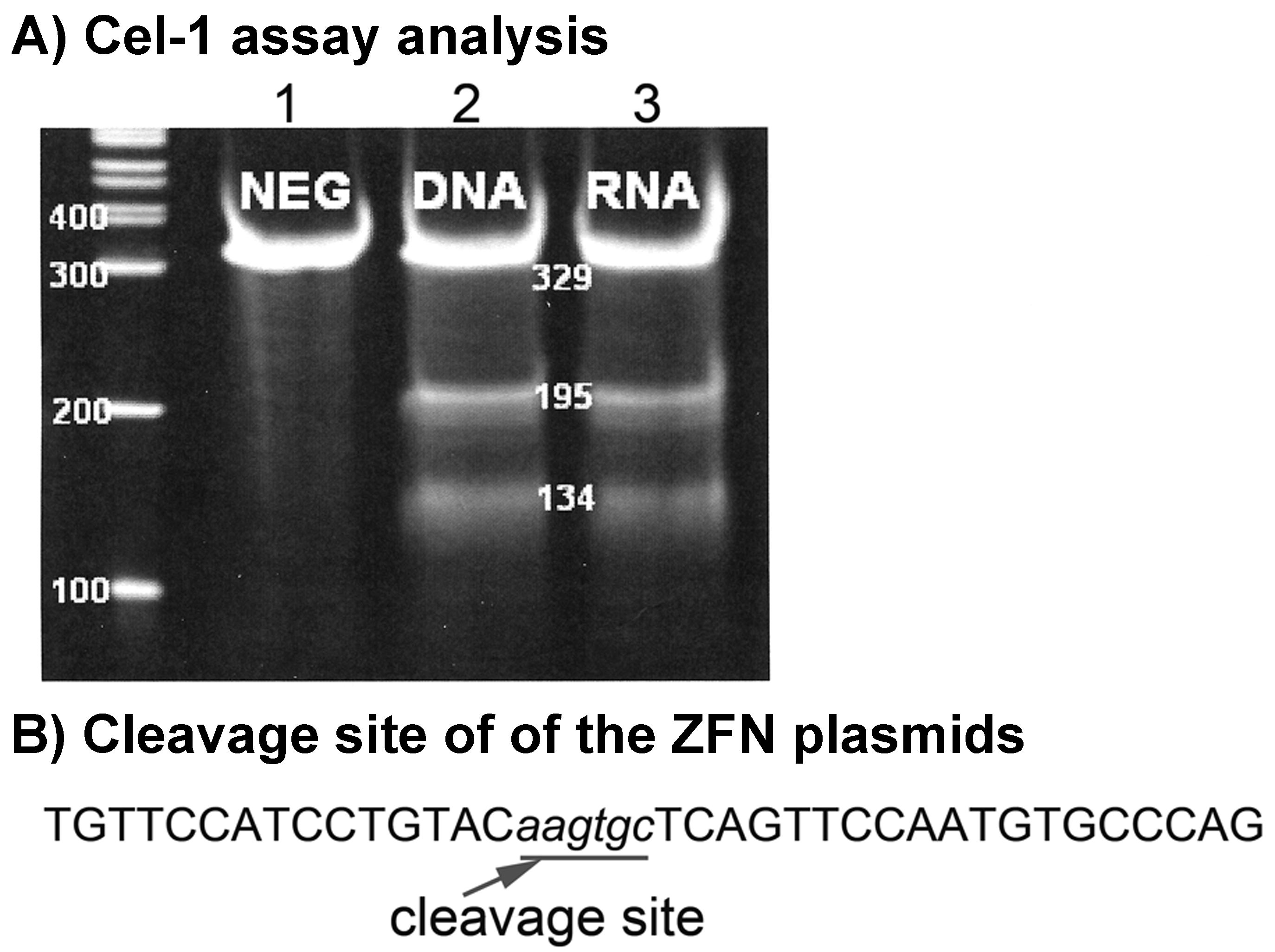

Supplement: S1 Fig — This assay was done by Sigma Aldrich to confirm the ZFN specificity. (A) DNA and RNA from untransfected and transfected cells were transfected with the left and right ZFN plasmids and grown for 2 days to allow random DNA repair. DNA and RNA samples were isolated. PCR was performed using a primer pair across the targeted cleavage site, and the PCR product, 329 bp, was treated with CEL-1, an endonuclease isolated from celery. CEL-1 has high specificity for mismatches, insertions, and deletions in DNA.CEL-1 mediated cleaved of the ZFN mutated PCR fragment generated two bands of 195 and 134 bp from the 329 bp fragment. (B) Cleavage site of the ZFNs located 59 bp from the TAA stop codon of the SNCA gene. (TIF) [file pone.0136930.s001.tif]

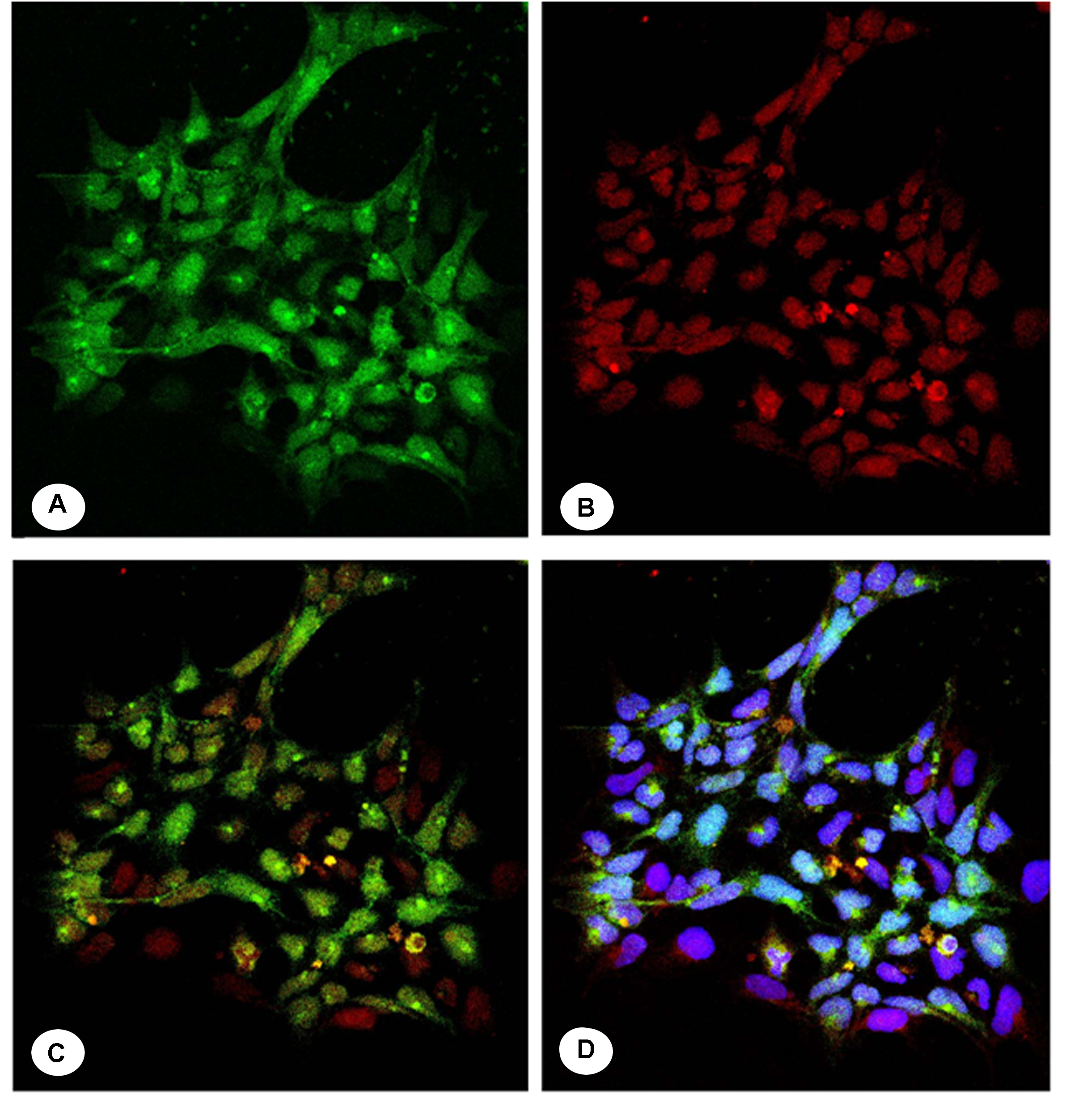

Supplement: S2 Fig — (A) GFP, (B) α-syn antibody, (C) overlay of GFP and anti-α-syn staining, (D) overlay of anti-GFP, anti-α-syn, and DAPI. The non-uniformity between GFP and α-synuclein labeling exists since the GFP12 cell line contains a mixed population of transfected cells. (TIF) [file pone.0136930.s002.tif]

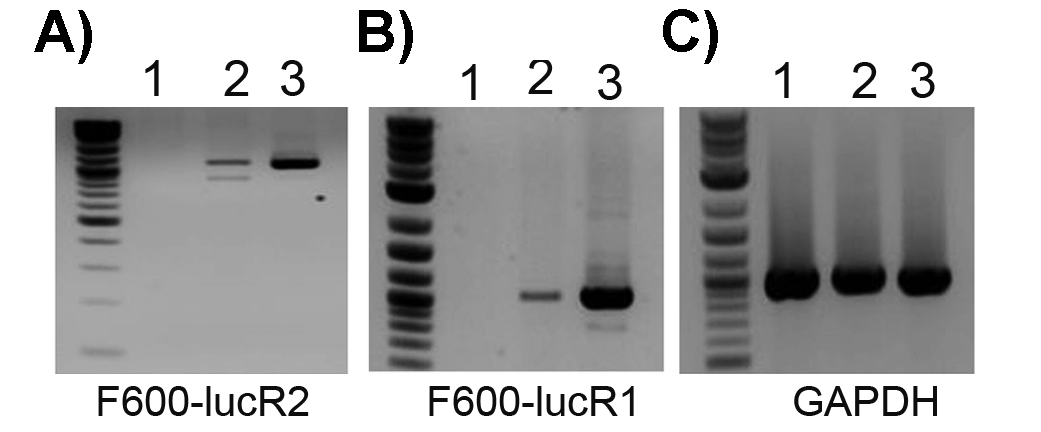

Supplement: S3 Fig — RT-PCR amplicons of RNAs isolated from Luc6B and Luc6B-5 cells using the F600/lucR1, F600/lucR2 and GAPDH primer pairs. Both primer pairs, F600/lucR1 and F600/lucR2, produced the correct bands at the predicted size for fragments generated by these primer pairs. Lane 1, SH-SY5Y, lane 2, Luc6B-5, and lane 3, Luc6B. These results showed that Luc6B cells expressed a high level of α-syn-luc mRNA than the Luc6B-5 cell line. Therefore, the Luc6B cell line was selected for detailed studies. (TIF) [file pone.0136930.s003.tif]

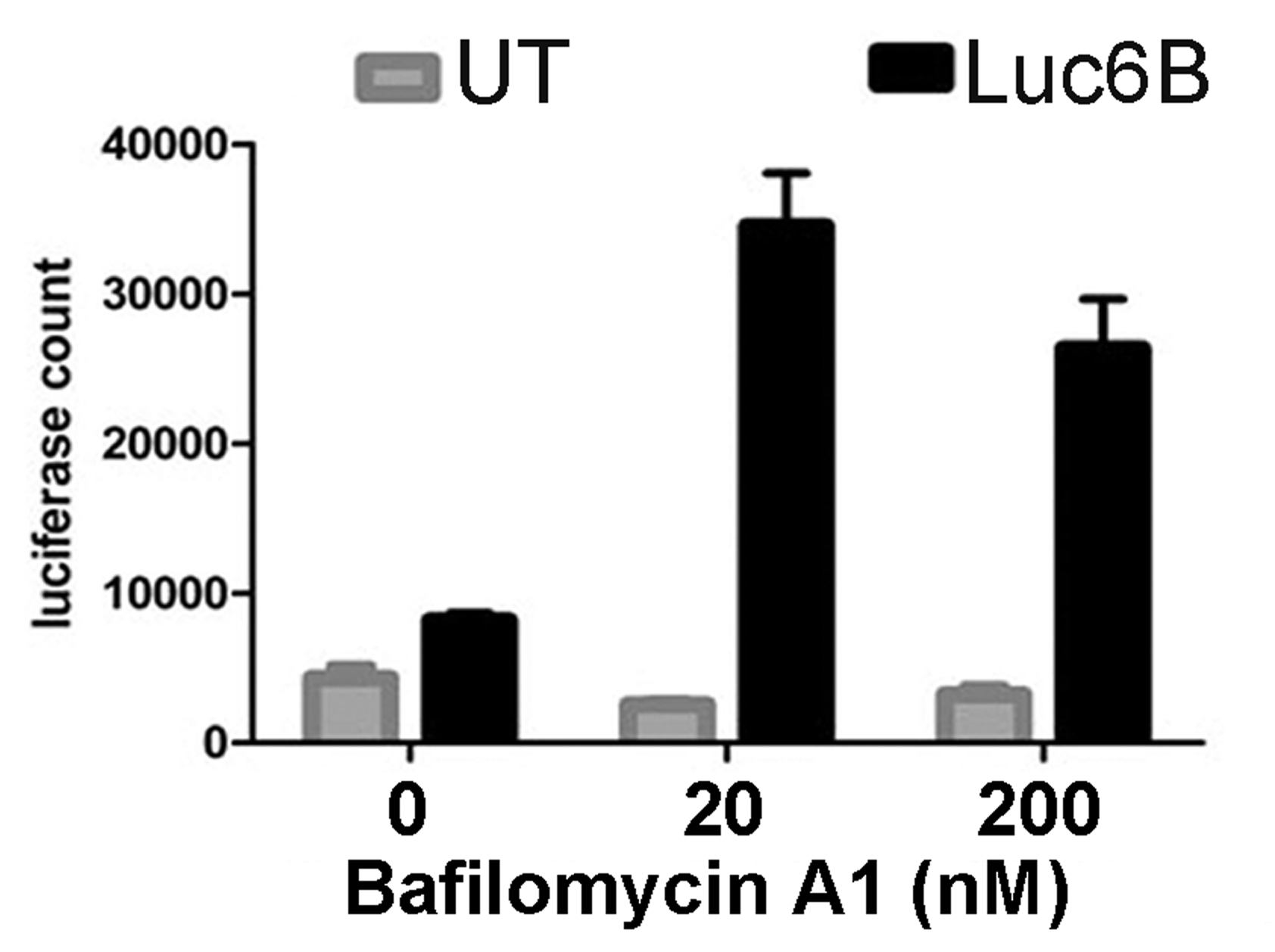

Supplement: S4 Fig — Bafilomycin A1 treatment increased the level of luciferase activities in Luc6B cells. SH-SY5Y (UT). and Luc6B cells were cultured in 6-well dishes, and grown in DMEM/FBS medium containing 50 μM retinoic acid for 8 days to differentiate cells into neuron-like cells. Cells were transferred to clean wells every 3–4 days. On the day prior to the experiment, cells were transferred to clean wells. The next day, cells were treated with DMSO, 20 nM, and 200 nM of bafilomycin A1. Luciferase activity was measured 24 hrs later using Promega Luciferase detection kit. Bafilomycin A1 was purchased from Sigma Aldrich. (TIF) [file pone.0136930.s004.tif]
